# Health-related quality of life and coping strategies among people living with HIV: the moderating role of gender

**DOI:** 10.1007/s00737-017-0801-2

**Published:** 2017-12-18

**Authors:** Marcin Rzeszutek

**Affiliations:** Faculty of Psychology, University of Warsaw, Stawki 5/7, 00-183 Warsaw, Poland

**Keywords:** HIV/AIDS, Health-related quality of life, Coping, Gender differences

## Abstract

The aim of the study was to explore gender differences in the level of health-related quality of life (HRQoL) and coping strategies among people living with the human immunodeficiency virus (HIV) (PLWH). In particular, the moderating role of participants’ gender on the relationship between coping strategies and HRQoL was explored, while controlling for socio-medical data. A total of 444 HIV-infected men and 86 HIV-infected women were recruited to participate in the study. This was a cross-sectional study with the HRQoL assessed by the World Health Organization (WHO) Quality of Life-BREF (WHOQOL-BREF) and the coping strategies measured by the Brief COPE inventory. Although the HIV-infected men and HIV-infected women differed in terms of some HRQoL domains, these differences disappeared in the regression analysis after controlling for socio-demographic data (employment and higher education). In addition, several statistically significant interactions between participants’ gender and coping strategies in relation to HRQoL domains were observed. Future research on gender differences in HRQoL among PLWH should take into account unique differences between HIV-infected men and HIV-infected women across, not only in respect to socio-medical factors but also regarding psychosocial variables.

Significant advances in the knowledge about and treatment of HIV infection has changed the nature of HIV/AIDS from a terminal disease to a chronic illness and has given people living with HIV (PLWH) new hope for increased life expectancy (Deeks et al. 2013; Samji et al. 2013). Nevertheless, PLWH still suffer from intense HIV-related distress, which stems not only from the awareness of a potentially lethal virus in their bodies, but also from the still existing stigma of HIV and the potential deterioration of their psychosocial status (Adewuya et al. 2009; Bogart et al. 2011; Machtinger et al. 2012; Pantalone et al. 2010; Rzeszutek et al. 2012, 2015). Therefore, apart from monitoring virological and immunological health outcomes, assessing subjective measures of health status, such as health-related quality of life (HRQoL), is becoming an integral part of medical examination among HIV/AIDS health services (Bucciardini et al. 2014; Burgoyne and Saunders 2001; Herrmann et al. 2013; Mitchell et al. 2017; Ruiz-Perez et al. 2005). However, although numerous studies have been conducted on factors associated with HRQoL among PLWH (Miners et al. 2014), they have not provided a coherent picture of what factors enhance HRQoL in this patient group (Degroote et al. 2014).

One topic that deserves more attention deals with gender differences in HRQoL among PLWH, which especially applies to HIV-infected women (Carvalhal 2010). Although the number of HIV-infected women has been constantly increasing globally (UNAIDS Report 2017), considerably more research has been conducted among HRQoL in HIV-infected men (Emuren et al. 2017; Jia et al. 2004; Liu et al. 2006; Song et al. 2016), compared with HIV-infected women (e.g., Gielen et al. 2001; McDonnell et al. 2000). When it comes to gender differences in HRQoL among PLWH, the majority of existing studies show a rather consistent trend, i.e., a lower level of HRQoL among HIV-infected women compared to HIV-infected men (Campsmith et al. 2003; Chandra et al. 2009; Mrus et al. 2005; Solomon et al. 2008). It must be mentioned that some authors observed poorer HRQoL among HIV-infected men (Peltzer and Phaswana-Mafuya 2008) or no gender differences in respect to HRQoL in this patient group (Ruiz-Perez et al. 2009). Various explanations for the poorer HRQoL among HIV-infected women were suggested, including unequal access to antiretroviral treatment (ART) in some countries for HIV-infected women (Penniman et al. 2007), a higher rate of physical and emotional abuse and mental disorders among HIV-infected women (Machtinger et al. 2012), especially the heightened HIV-related stigma among HIV-infected women (Geary et al. 2014) which prevents them from disclosing their HIV+ status and seeking medical care (Campbell et al. 2006). However, other authors noticed significant gender differences in clinical outcomes, i.e., HIV-infected women reported higher CD4 count, lower viral load, and better adherence to treatment compared to HIV-infected men (Collazos et al. 2007; Nicastri et al. 2007). The nature of gender differences in HRQoL is complex, but, according to the newest studies, these differences may depend to a greater extent on socio-demographic or psychosocial factors rather than on the objective health status (Degroote et al. 2014; Vo et al. 2016).

One of these important psychosocial factors is coping with HIV infection (Gore-Felton et al. 2002; Vosvick et al. 2010). Although a significant number of studies were conducted on coping among PLWH (see, Moskowitz et al. 2009), several authors used different coping tools and relied on various coping classifications. This precludes obtaining an unambiguous picture of coping effectiveness among PLWH. Nevertheless, some agreement exists regarding three ways of coping: active coping, avoidance coping, and meaning-focused coping. Active coping appeared to be positively related not only to HIV-related clinical outcomes, but also to the enhancement of HRQoL among PLWH (Chida and Vedhara 2009; Ironson and Hayward 2008; Pence et al. 2008). Different forms of avoidance coping, including behavioral disengagement, substance use, and social isolation predicted consequent deterioration of health status, lower HRQoL, and lower adherence to treatment regimens (Griswold et al. 2005; Vervoort et al. 2009). Finally, meaning-focused coping appeared to be positively associated with affective well-being (Moskowitz et al. 2009, 2012). Regarding gender differences in coping with HIV infection, the results are mixed, ranging from studies that highlighted the lack of gender differences (Ashton et al. 2005; Gore-Felton et al. 2002) to research that pointed to a higher intensity of avoidance coping, spiritual coping, and seeking social support as coping strategies among HIV-infected women (Tarakeshwar et al. 2005; Vosvick et al. 2002). What remains especially unclear are gender differences in coping effectiveness, i.e., whether and how specific coping strategies may be related to selected aspects of physical, psychological, and social functioning separately among HIV-infected men and HIV-infected women.

## Current study

The aim of the current study was to explore gender differences in the level of HRQoL and coping strategies among people living with HIV. In particular, the moderating role of participants’ gender on the relationship between coping strategies and HRQoL was explored, while controlling for socio-medical data. Based on the literature review, one direct and one indirect hypothesis were formulated; however, in this last hypothesis, an exploratory approach has primarily been utilized due to the lack of studies on the aforementioned topic. Firstly, it was expected that HIV-infected women would score lower on particular dimensions of HRQoL compared to HIV-infected men. Secondly, it was expected that the coping strategies would be differently related to particular HRQoL domains among HIV-infected women and HIV-infected men.

## Method

### Participants

A total of 444 HIV-infected men and 86 HIV-infected women were recruited from the patients of the outpatient clinic of the state hospital for infectious diseases. Participants completed a paper-and-pencil version of the two designated measures and participated voluntarily in the study; there was no remuneration. The eligibility criteria were that participants had to be 18 years of age or older, medically diagnosed as HIV-infected and receiving treatment from the hospital where the study was conducted. The exclusion criteria included HIV-related cognitive impairment diagnosed by medical doctors. This research project was also accepted by the local ethics committee. Socio-medical data characteristics among HIV-infected men and HIV-infected women are included in Table 1.

**Table 1:** Socio-medical variables in the studied sample of HIV-infected men (*N* = 444) and HIV-infected women (*N* = 86)

Variable	Male	Female	*p*
	*N* (%)	*N* (%)	
Age in years (M ± SD)	39.75 ± 10.54	40.09 ± 10.60	*t*(528) = − .27, *p* > .05
Marital status			
Married	245 (55.2%)	58 (67.4%)	*χ* ^2^(1) = 4.42. *p* < .05
Single	199 (44.8%)	28 (32.6%)	
Education			
Elementary	22 (5.0%)	18 (20.9%)	*χ* ^2^(3) = 32.76. *p* < .001
Occupational	38 (8.6%)	6 (7.0%)	
Secondary	130 (29.3%)	32 (37.2%)	
University degree	254 (57.2%)	30 (34.9%)	
Employment			
Full employment	339 (76.4%)	45 (52.3%)	*χ* ^2^(3) = 3.71. *p* < .001
Unemployment	42 (9.5%)	19 (22.1%)	
Retirement	20 (4.5%)	1 (1.2%)	
Sickness allowance	43 (9.7%)	21 (24.4%)	
HIV/AIDS status			
HIV+ only	375 (84.5%)	73 (84.9%)	*χ* ^2^(1) = .01. *p* > .05
HIV/AIDS	69 (15.5%)	13 (15.1%)	
HIV infection duration in years (M ± SD)	7.17 ± 6.48	10.86 ± 7.88	*t*(528) = − 4.08, *p* < .001
Antiretroviral treatment (ART) duration in years (M ± SD)	5.48 ± 5.17	8.53 ± 6.57	*t*(528) = − 4.79, *p* < .001
CD4 Count	587.87 ± 223.13	597.64 ± 219.79	*t*(528) = − .37, *p* > .05

*M*, mean; *SD*, standard deviation; *t*, value of independent samples *t* test; *χ* ^2^, Pearson’s Chi-squared test; *p*, statistical significance

### Measures

Health-related quality of life was assessed with the use of the World Health Organization (WHO) Quality of Life-BREF (WHOQOL-BREF), created by the WHO initiative to assess this construct cross-culturally (WHOQOL Group 1995) in a Polish adaptation. The WHOQOL-BREF consisted of 26 items to measure four domains: somatic health, psychological health, social relationships, and environment. Cronbach’s alpha coefficients for the current study were satisfactory and exceeded .86 for the above-mentioned HRQoL domains.

To measure strategies for coping with stress, the Brief COPE Inventory was used (Carver and Scheier 1997) in a Polish adaptation. The questionnaire included 28 items and provided 14 subscales, two items each, with a Likert-like response scale ranging from 0 (I have not been doing this at all.) to 3 (I have been doing this a lot.). The aforementioned subscales were derived empirically, and they were not theoretically reassessed afterwards to obtain a more comprehensive systematization of coping strategies (Skinner et al. 2003). More specifically, as this tool does not contain items directly describing rumination, which is one of the most strongly proven maladaptive strategies (see Nolen-Hoeksema et al. 2008) and items describing coping efforts to enhance positive emotions during stressful situations, these two items were added from the Ruminative Response Styles (Treynor et al. 2003): I think “What am I doing to deserve this?”; I think “Why do I have problems other people don’t have?”) and from the Coping with Health Injuries and Problems Scale after modification (Endler et al. 1998): I have nice things around; I look for simple pleasures. Finally, 16 coping strategies were studied: self-distraction, active coping, denial, substance use, use of emotional support, use of instrumental support, behavioral disengagement, venting, positive reframing, planning, humor, acceptance, religion, self-blame, rumination, and positive emotion enhancement. The Cronbach’s alpha coefficients for the current study were satisfactory and ranged from .78 to .86 for all subscales.

### Data analysis

The process of statistical analysis consisted of three steps. Firstly, the group of HIV-infected women and the group of HIV-infected men were compared in terms of socio-medical data. Secondly, these two groups were compared in terms of HRQoL and coping strategies. Thirdly, in order to verify if participants’ gender moderates the relationship between coping strategies and HRQoL, regression analysis was performed. The statistical analysis was performed using the IBM SPSS Statistics 24 software released in 2016 (IBM Corp. Released 2016).

## Results

Analyzed variables did not differ from the normal distribution, i.e., values of skewness for analyzed variables ranged from − .81 to .26, and kurtosis values or analyzed variables ranged from − .71 to .76. Table 2 presents gender differences in terms of HRQoL domains and coping strategies.

**Table 2.** Gender differences in health-related quality of life and of coping strategies.

Variable	Male	Female	*t*	df	*p*
	*M* (SD)	*M* (SD)			
WHO_somatic	25.48 (4.95)	24.06 (5.36)	2.40	528	.017
WHO_psychological	22.23 (4.24)	21.56 (4.27)	1.33	528	.183
WHO_social	11.08 (2.38)	11.22 (2.30)	− .49	528	.621
WHO_environmental	30.46 (5.29)	28.50 (5.65)	3.11	528	.002
Active coping	3.46 (1.26)	3.48 (1.22)	− .10	528	.919
Planning	3.60 (1.34)	3.59 (1.35)	.05	528	.958
Positive reframing	3.54 (1.30)	3.60 (1.29)	− .45	528	.654
Acceptance	3.95 (1.20)	3.81 (1.13)	.97	528	.330
Humor	2.87 (1.42)	2.64 (1.34)	1.39	528	.166
Religion	2.40 (1.90)	2.41 (1.76)	− .03	528	.978
Use of emotional support	2.68 (1.58)	2.72 (1.52)	− .21	528	.835
Use of instrumental support	3.39 (1.34)	3.49 (1.42)	− .63	528	.527
Self-distraction	2.98 (1.33)	3.17 (1.10)	− 1.25	528	.213
Denial	2.22 (1.63)	2.26 (1.55)	− .20	528	.845
Venting	2.93 (1.41)	2.97 (1.49)	− .22	528	.825
Substance use	2.25 (1.84)	1.83 (1.71)	1.97	528	.050
Behavioral disengagement	2.35 (1.62)	2.21 (1.61)	.76	528	.448
Self-blame	2.88 (1.58)	2.67 (1.54)	1.10	528	.272
Rumination	2.76 (1.74)	2.74 (1.78)	.09	528	.925
Positive emotion enhancement	3.91 (1.36)	3.86 (1.38)	.28	528	.779

*M*, mean; *SD*, standard deviation; *t*, value of independent samples *t* test; *df*, degrees of freedom; *p*, statistical significance

The main statistical method used was hierarchical regression analysis (Darlington and Hayes 2017). The first block was devoted to the selection using stepwise method of socio-medical variables that were significantly related to HRQoL domains, and, therefore, should be controlled. The second block added the main effects of gender and coping. The final block added the interaction between gender and coping. Table 3 presents results for the models with statistically significant interactions. Each interaction was followed by a simple effects analysis, which was performed separately in the groups of HIV-infected men and HIV-infected women.

**Table 3:** Statistically significant interactions between participants’ gender and coping strategies in relation to health-related qualify of life domains

Outcome	Predictors	Total model				Simple effects analysis					
		Beta	*t*	*p*	*ΔR* ^2^	Predictor	Moderator	Beta	*t*	*p*	*ΔR* ^2^
WHO_somatic	Employment	.32	7.55	.001	0.10	Positive reframing					
	Gender	− .05	− 1.09	.278	0.01		Male	.01	.32	.752	0.01
	Positive reframing	.14	2.43	.016	0.01		Female	.24	2.36	.020	0.06
	Positive reframing × gender	.12	2.19	.029	0.01						
WHO_somatic	Employment	.30	7.30	.001	0.10	Use of emotional support					
	Gender	− .04	− 1.07	.283	0.01		Male	− .15	− 3.39	.001	.020
	Use of emotional support	.02	.27	.788	0.01		Female	.16	1.55	.124	.030
	Use of emotional support × gender	.16	2.88	.004	0.01						
WHO_environmental	Employment	.26	5.75	.000	0.08	Positive reframing					
	Higher education	.17	3.98	.001	0.03						
	Age	.12	2.84	.005	0.02						
	CD4	.09	2.28	.023	0.01						
	Gender	− .06	− 1.48	.140	0.01		Male	− .03	− .58	.566	.010
	Positive reframing	.11	1.92	.050	0.01		Female	.26	2.58	.012	.070
	Positive reframing × gender	.13	2.41	.016	0.01						
WHO_social	Stable relationship	− .21	− 5.07	.000	0.06	Use of emotional support					
	Employment	.15	3.27	.001	0.02						
	AIDS	− .11	− 2.56	.011	0.01						
	Higher education	.09	1.96	.050	0.01						
	Gender	.04	1.03	.303	0.01		Male	− .02	− .43	.671	.010
	Use of emotional support	.13	2.18	.030	0.01		Female	.29	2.77	.007	.080
	Use of emotional support × gender	.15	2.51	.012	0.01						
WHO_psychological	Employment	.26	5.79	.000	0.06	Positive emotion enhancement					
	CD4	.14	3.50	.001	0.02						
	Stable relationship	− .11	− 2.68	.008	0.01						
	Age	.10	2.24	.026	0.01						
	Gender	− .02	− .39	.699	0.01		Male	.07	1.49	.138	.010
	Positive emotion enhancement	.25	4.61	.000	0.02		Female	.40	3.81	.001	.140
	Positive emotion enhancement × gender	.18	3.31	.001	0.02						

*Beta*, standardized regression coefficients; *t*, test for significance of regression coefficient; *p*, statistical significance; *ΔR* ^2^, change of determination coefficient

None of the main of effects of gender was statistically significant, despite the statistically significant differences between means noticed in Table 2. HIV-infected men and HIV-infected women differed not only in terms of mean values of HRQoL but also in terms of socio-medical data which were significantly related to HRQoL domains. After inclusion of these data in the regression model, the effect of gender was statistically insignificant. There were no statistically significant differences in HRQoL domains that could be attributed directly to gender when gender differences regarding employment and higher education were taken into consideration.

**Fig. 1:**
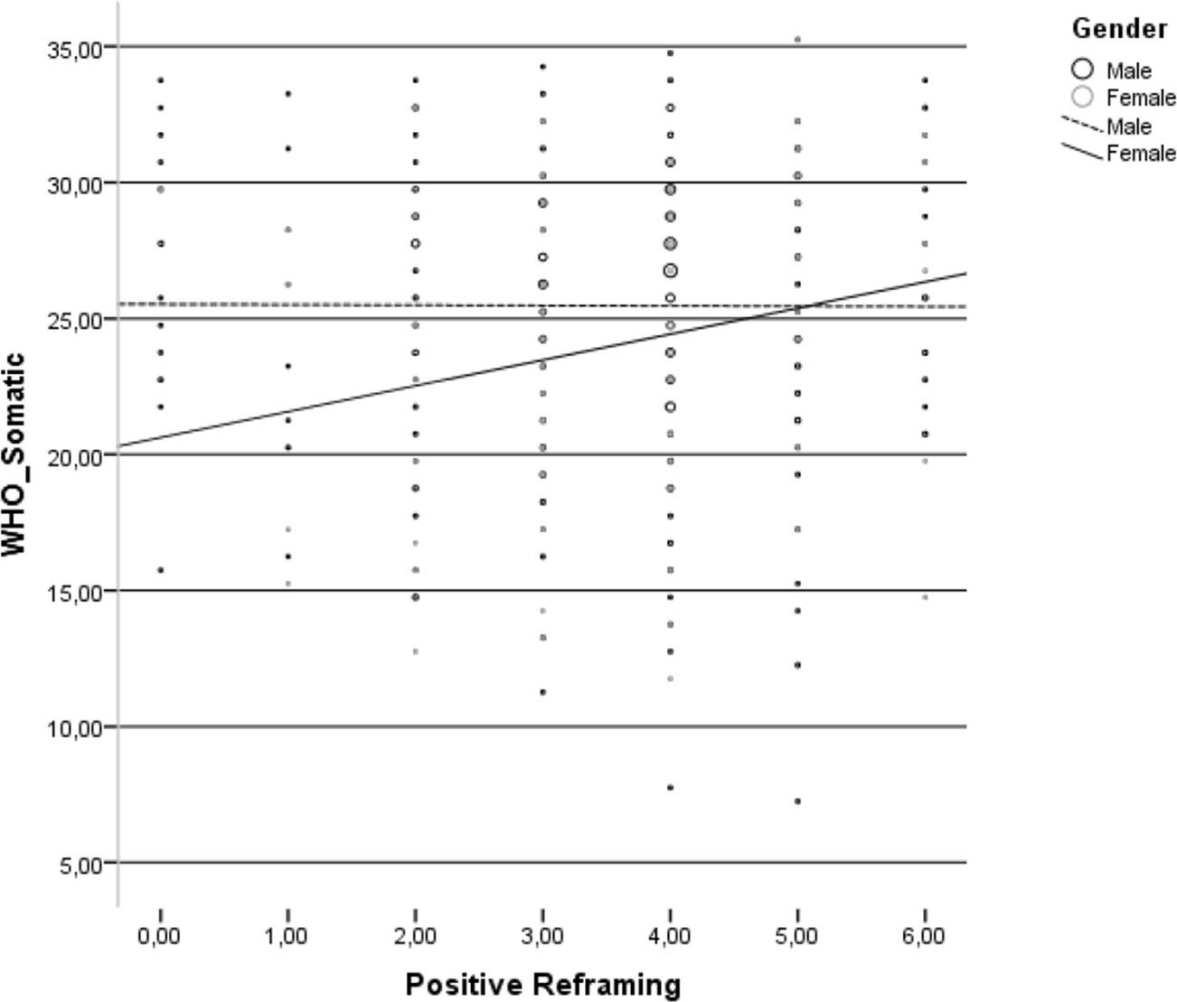
Relationship between positive reframing and somatic domain of health-related quality of life in the group of HIV-infected men and HIV-infected women

There were five statistically significant interactions between participants’ gender and coping strategies in relation to HRQoL domains. Positive reframing was positively related to the HRQoL somatic domain only in the group of HIV-infected women (Fig 1).

**Fig. 2:**
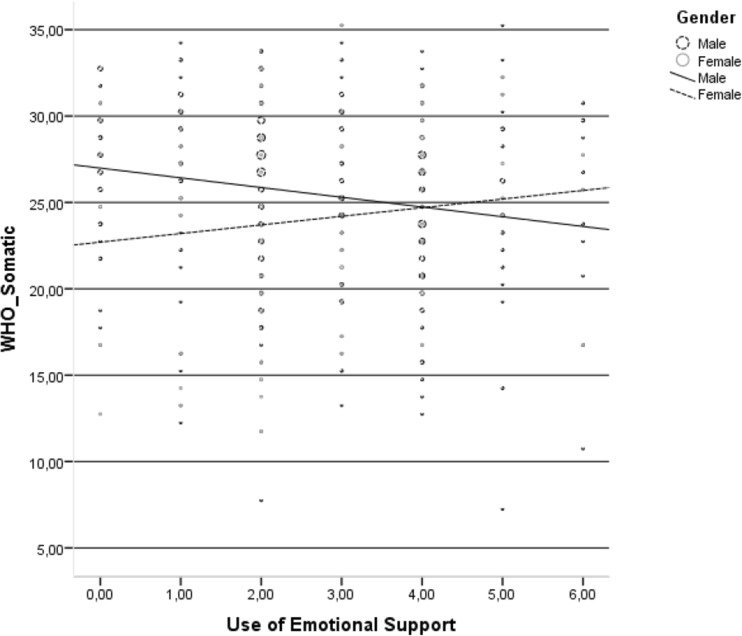
Relationship between use of emotional support and somatic domain of health-related quality of life in the group of HIV-infected men and HIV-infected women

Use of emotional support was negatively related to the HRQoL somatic domain only in the group HIV-infected men (Fig 2).

**Fig. 3:**
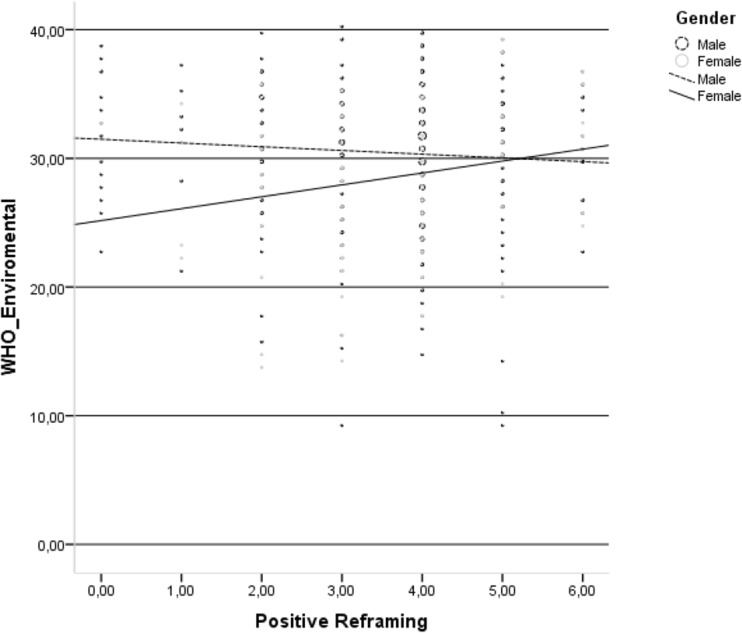
Relationship between positive reframing and environmental domain of health-related quality of life in the group of HIV-infected men and HIV-infected women

Positive reframing was positively related to the HRQoL environmental domain only in the group of HIV-infected women (Fig. 3).

**Fig. 4:**
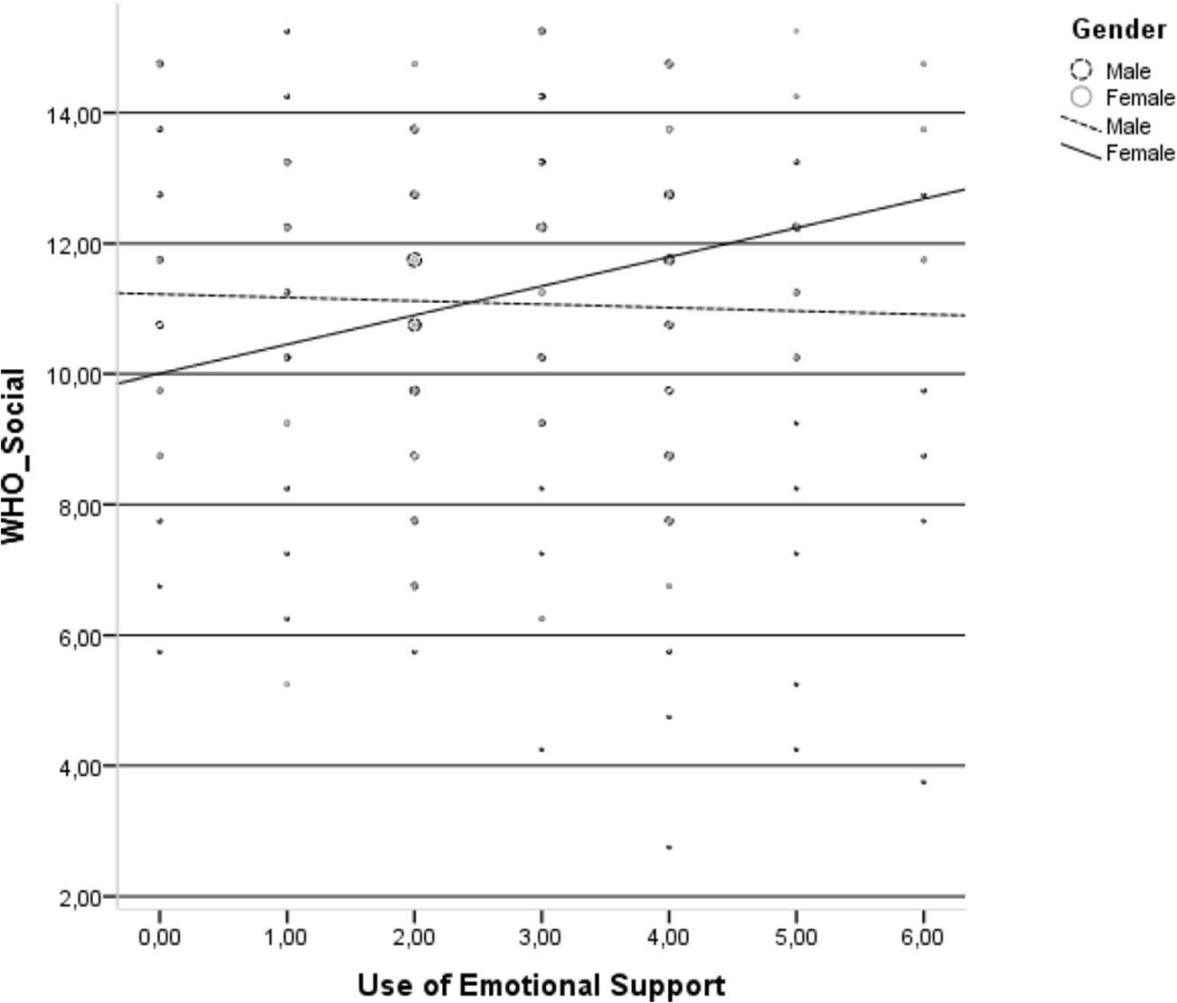
Relationship between use of emotional support and social domain of health-related quality of life in the group of HIV-infected men and HIV-infected women

Use of emotional support was positively related to the HRQoL social domain only in the group of HIV-infected women (Fig. 4.).

**Fig. 5:**
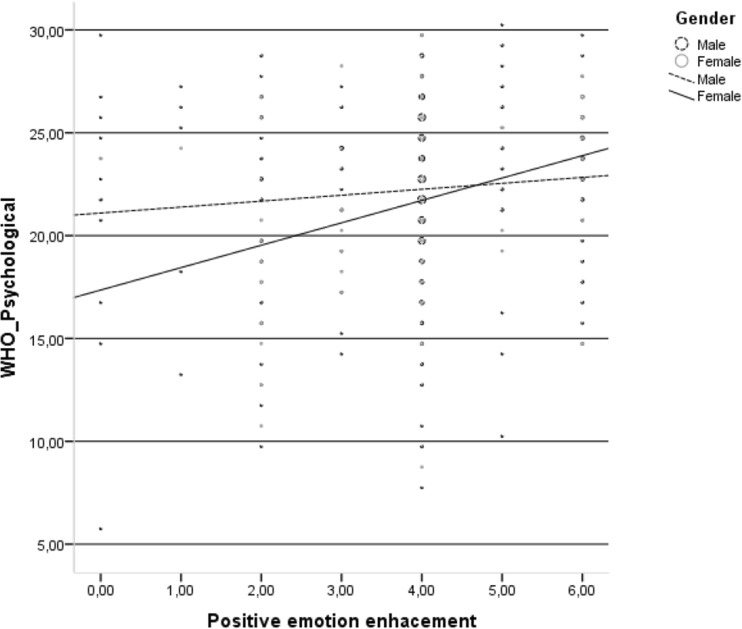
Relationship between positive emotion enhancement and psychological domain of health-related quality of life in the group of HIV-infected men and HIV-infected women

Finally, positive emotion enhancement was positively related to the HRQoL psychological domain only in the group of HIV-infected women (Fig. 5).

## Discussion

The results of this study were somewhat inconsistent with the first hypothesis, i.e., although HIV-infected women reported a lower level of particular HRQoL domains, these differences disappeared in the regression analysis after controlling for socio-demographic data (employment and higher education). These findings contradict the aforementioned studies, which showed lower levels of HRQoL among HIV-infected women (Campsmith et al. 2003; Mrus et al. 2005; Solomon et al. 2008). However, Ruiz-Perez et al. (2009) also observed that the gender difference in HRQoL among PLWH disappeared after adjusting the results in terms of demographic and clinical data. In this study, the lower level of HRQoL in the somatic and environmental domains among HIV-infected women can be understood rather as a derivative of a worse occupational situation and a lower level of education compared to HIV-infected men. On one hand, several authors observed the beneficial role of employment on HRQoL among PLWH, even after controlling for the severity of HIV progression (Blalock et al. 2002; Worthington and Krentz 2005). More recently, Ruedaa et al. (2011) showed that employment status was the strongest predictor of physical and mental domain of HRQoL among PLWH and outweighed the role of other demographic factors, social support, clinical variables, and even neurocognitive functioning. It is well-known that PLWH are under constant threat of losing their employment (Samson et al. 2009), and, when they lose their jobs, they face several barriers to workforce re-entry (Martin et al. 2003); this especially relates to HIV-infected women (Aziz and Smith 2011). Similarly, higher education appeared to be not only a personal resource in coping with HIV-related distress among PLWH (O’Leary et al. 2014) and also protected them from depression (Lee et al. 2014), but it was also positively related to HRQoL (da Silva et al. 2013). Conversely, lower education is significantly correlated to heightening perceived stigma, observed especially among HIV-infected women (Wagner et al. 2010).

The second hypothesis was positively verified, as several statistically significant interactions between participants’ gender and coping strategies in relation to HRQoL domains were observed. Interestingly, in almost all instances of these interactions, the relationship between HRQoL and coping was statistically significant in the group of HIV-infected women, which may indicate that gender differences in HRQoL among PLWH are strictly associated with appropriate coping with HIV infection. More specifically, positive reframing was positively related to the somatic and environmental domains of HRQoL only in the group of HIV-infected women. Until now, positive reframing appeared to be positively related to different domains of QoL among PLWH in a few studies, but gender differences were not taken into account (Armon and Lichtenstein 2012; Moskowitz et al. 2009, 2012). In only one research that of Weaver et al. (2004), was it found that HIV-infected women who relied more on positive reframing were characterized by higher levels of various QoL domains as they perceived lower levels of stress in their closest environment.

Additionally, whereas the use of emotional support appeared to be negatively associated with the somatic domain of HRQoL in the group of HIV-infected men, this coping strategy was positively linked with the HRQoL social domain in the group of HIV-infected women. This specific finding was somewhat opposed to that of other studies, which showed that HIV-infected men gain more in terms of well-being from receiving emotional support compared to HIV-infected women, as the latter have difficulty in accepting this type of support due to the greater level of HIV-related stigma that they experience (Gordillo et al. 2009). On the other hand, Rzeszutek et al. (2016) observed that HIV-infected women had a higher need for support and, thus, received more support compared to HIV-infected men. This is also in accordance with other studies that pointed to a high intensity of support seeking among HIV-infected women, despite stigmatization (Jones et al. 2007). Furthermore, Kotze et al. (2013) longitudinal research found that an increase in active coping was positively related with receiving more social support among newly HIV-infected women during pregnancy which, in turn, resulted in a decrease in perceived stigma. Thus, one may hypothesize that, among HIV-infected women, appropriate coping may play a beneficial role in various aspects of social functioning, including managing HIV-related stigma and various barriers to seeking and receiving social support (Vyavaharkar et al. 2007).

Finally, positive emotional enhancement was positively related to the psychological domain of HRQoL only in the group of HIV-infected women. An increasing number of authors highlighted the beneficial role of positive effects among PLWH in various areas of functioning (Ironson and Hayward 2008; Moskowitz et al. 2017). More specifically, positive effects were negatively related to mortality rate (Moskowitz 2003), predicted lower viral load (Wilson et al. 2016), caused slower progression of HIV infection (Ironson and Hayward 2008) and were negatively associated with depression (Li et al. 2016). In terms of specific positive affect actions, interventions appeared to be successful in improving psychological functioning and adjustment among PLWH (Moskowitz et al. 2017); thus, these types of interventions should be especially tailored to HIV-infected women.

## Limitations

This study is not free from limitations. Firstly, the cross-sectional design of this study precludes causal interpretations. It specifically deals with understanding the relationship between HRQoL and coping strategies. Secondly, other socio-demographic (e.g., sexual orientation) or clinical variables (e.g., HIV transmission and viral load) were not controlled in this study. Finally, a significant underrepresentation of women may be observed in the study, but the gender ratio was rather typical for other studies conducted on PLWH (Bor et al. 2015).

## Conclusions

The results of this study suggest that future research on the aforementioned topic should take into account the unique differences between HIV-infected men and HIV-infected women across not only socio-medical factors, but also in regard to psychosocial variables. Perhaps, more complex controlling of these differences may verify the traditional trend in the literature pointing to lower HRQoL among HIV-infected women. More specifically, it seems that HRQoL among HIV-infected women depends more on coping strategies compared to HIV-infected men; this should be taken into account in the psychological counseling of HIV-infected women.
